# Evaluating the In Vivo Specificity of [^18^F]UCB-H for the SV2A Protein, Compared with SV2B and SV2C in Rats Using microPET

**DOI:** 10.3390/molecules24091705

**Published:** 2019-05-01

**Authors:** Maria Elisa Serrano, Guillaume Becker, Mohamed Ali Bahri, Alain Seret, Nathalie Mestdagh, Joël Mercier, Frédéric Mievis, Fabrice Giacomelli, Christian Lemaire, Eric Salmon, André Luxen, Alain Plenevaux

**Affiliations:** 1 GIGA—CRC In Vivo Imaging, University of Liège, 8 Allée du 6 Août, Building B30, Sart Tilman, 4000 Liège, Belgium; meserrano@uliege.be (M.E.S.); guillaume.becker@sckcen.be (G.B.); M.Bahri@uliege.be (M.A.B.); aseret@uliege.be (A.S.); Christian.Lemaire@uliege.be (C.L.); Eric.Salmon@uliege.be (E.S.); aluxen@uliege.be (A.L.); 2 UCB Pharma s.a., 1420 Braine-l’Alleud, Belgium; Nathalie.Mestdagh@ucb.com (N.M.); joel.mercier@ucb.com (J.M.); 3 Nucleis s.a., University of Liège, 8 Allée du 6 Août, Building B30, Sart Tilman, 4000 Liège, Belgium; frederic.mievis@nucleis.eu (F.M.); fabrice.giacomelli@nucleis.eu (F.G.)

**Keywords:** SV2A, SV2B, SV2C, microPET, [^18^F]UCB-H, epilepsy, PBIF, distribution volume, blocking assay, preclinical imaging

## Abstract

The synaptic vesicle protein 2 (SV2) is involved in synaptic vesicle trafficking. The SV2A isoform is the most studied and its implication in epilepsy therapy led to the development of the first SV2A PET radiotracer [^18^F]UCB-H. The objective of this study was to evaluate in vivo, using microPET in rats, the specificity of [^18^F]UCB-H for SV2 isoform A in comparison with the other two isoforms (B and C) through a blocking assay. Twenty Sprague Dawley rats were pre-treated either with the vehicle, or with specific competitors against SV2A (levetiracetam), SV2B (UCB5203) and SV2C (UCB0949). The distribution volume (Vt, Logan plot, t* 15 min) was obtained with a population-based input function. The Vt analysis for the entire brain showed statistically significant differences between the levetiracetam group and the other groups (*p* < 0.001), but also between the vehicle and the SV2B group (*p* < 0.05). An in-depth Vt analysis conducted for eight relevant brain structures confirmed the statistically significant differences between the levetiracetam group and the other groups (*p* < 0.001) and highlighted the superior and the inferior colliculi along with the cortex as regions also displaying statistically significant differences between the vehicle and SV2B groups (*p* < 0.05). These results emphasize the in vivo specificity of [^18^F]UCB-H for SV2A against SV2B and SV2C, confirming that [^18^F]UCB-H is a suitable radiotracer for in vivo imaging of the SV2A proteins with PET.

## 1. Introduction

The synaptic vesicle protein 2 (SV2) is an integral membrane protein with twelve transmembrane domains and three N-glycosylation sites in the intravesicular loop. The SV2 protein is ubiquitously present in the nerve terminals of the central and peripheral nervous systems, and in several types of endocrine cells [1]. This protein is critical for the adequate functioning of the central nervous system, acting as a modulator of synaptic transmission [2,3]. Moreover, it has been associated with the pathophysiology of epilepsy [4,5,6].

Previous studies have identified three SV2 isoforms: SV2A, SV2B and SV2C, characterized by different expression levels during rodent brain development [7] and adulthood [8]. While the SV2A isoform is present across all brain areas, the SV2C isoform can only be found in specific regions, such as the striatum, pallidum, midbrain, brainstem, substantia nigra, and the olfactory bulb [9]. The SV2B isoform is particularly present in the cerebral cortex, and the cornu ammonis sub-region of the hippocampus [10]. The three isoforms present large similarities in their structure: 65% between isoforms A and B, 62% between A and C, and 57% between B and C [8].

Of these three isoforms, SV2A is the most investigated. The antiepileptic drug levetiracetam (Keppra^®^) binds to SV2A, suggesting a role for SV2A in the pathology underlying certain forms of epilepsy [11,12,13,14]. Several studies have shown a correlation between the brain expression of this isoform and the clinical efficacy of this drug [5,13].

**Figure 1:**
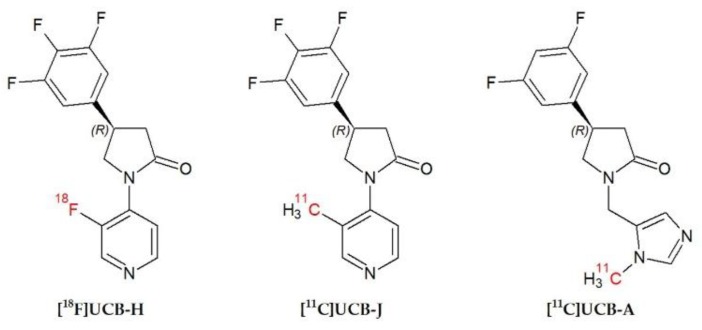
Chemical structures of [^18^F]UCB-H, [^11^C]UCB-J and [^11^C]UCB-A.

To investigate the role of SV2A in vivo, in 2013 [^18^F]UCB-H was presented as an imaging agent with a nanomolar affinity for human SV2A [3,15,16,17]. Since then, other PET radiotracers, such as [^11^C]UCB-J, or [^11^C]UCB-A, have been synthetized to study this protein [3,18,19,20] (see Figure 1). These PET radiotracers appear to be more specific than [^18^F]UCB-H (pIC_50_ = 7.8) [3,16], based on their respective affinity measured in vitro, with pIC_50_ = 8.2 for [^11^C]UCB-J [18] and pIC_50_ = 7.9 for [^11^C]UCB-A [20]. The three radiotracers have demonstrated potential for use as synaptic density biomarkers not only in animals, but also in humans [3,21,22,23]. However, despite the valuable properties of [^11^C]UCB-J and [^11^C]UCB-A in assessing brain synaptic density in vivo, their clinical application is limited to facilities with a cyclotron due to the short half-life of ^11^C (20.3 min) compared to the half-life of ^18^F (110 min). In addition, the use of a PET radiotracer with a longer half-life (such as ^18^F) allows the evaluation of a greater number of patients per day with just one production. Therefore, different fluorine-18-labelled derivatives of UCB-J are currently being developed and characterized, such as [^18^F]SDM-8 [24]. The potential of [^18^F]UCB-H for detecting variations in SV2A has already been demonstrated in vivo [25,26]. Nevertheless, as the actual specificity of [^18^F]UCB-H for SV2A against SV2B and SV2C has never been addressed in vivo, we consider that this point deserves more careful evaluation.

This paper, therefore, aims to evaluate for the first time the specificity of [^18^F]UCB-H for the SV2A isoform against SV2B and SV2C using microPET imaging in rats, by means of a blocking assay between this radiotracer and specific competitors for the three SV2 isoforms. The results will provide highly valuable information about the actual potential of [^18^F]UCB-H as a radiopharmaceutical candidate to study the SV2A protein with PET in research or clinical practice.

## 2. Results

Table 1 summarizes the results obtained from the in vitro binding assays. We observe that SV2B_L_ presents a high affinity for SV2B (pIC_50_ = 7.8), but also has an affinity for SV2A similar to that of SV2A_L_ (pIC_50_ = 5.6).

**Figure 2:**
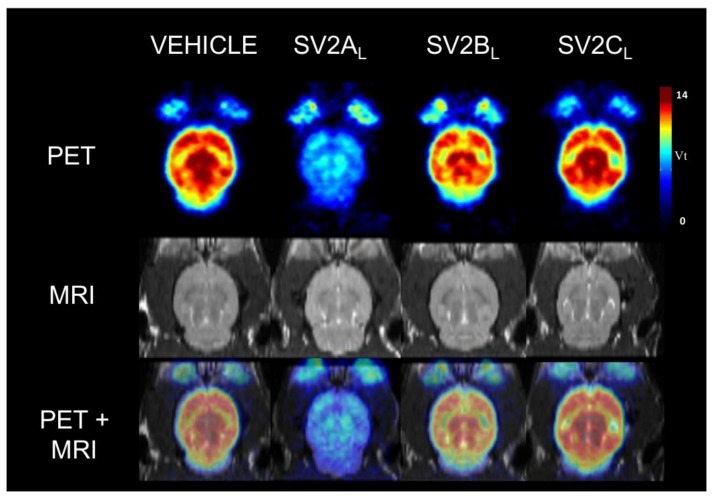
Example of an individual parametric Vt map of [^18^F]UCB-H binding in rat brain (PET), along with the corresponding individual MRI and the overlay of both images (PET + MRI). Rats were pre-treated 30 min before the 60 min PET acquisition with either vehicle, SV2A competitor (levetiracetam [SV2A_L_] at 10 mg/kg), SV2B competitor (UCB5203 [SV2B_L_] at 3 mg/kg) and SV2C competitor (UCB0949 [SV2C_L_] at 3 mg/kg).

In Figure 2, [^18^F]UCB-H parametric Vt maps are presented for the vehicle group and the three pre-treated groups (SV2A_L_, SV2B_L_ and SV2C_L_). These pictures highlight a clear reduction of the [^18^F]UCB-H binding throughout the entire brain induced by levetiracetam (SV2A_L_) pre-treatment at 10 mg/kg (PET image corresponding to the SV2A_L_ group).

**Figure 3:**
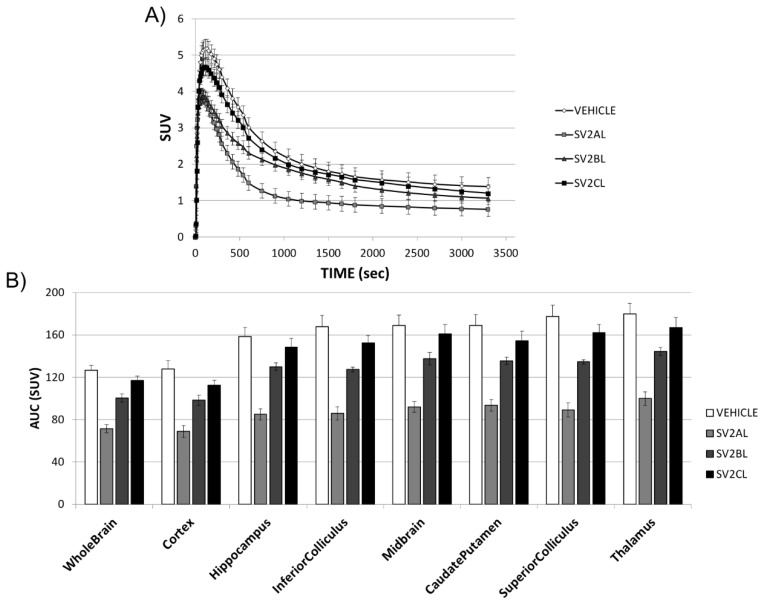
Representative time activity curves (TACs) and AUC (area under each TAC) for the different regions of interest (ROIs). (**A**) TACs extracted from the whole brain as ROI, and normalized by the injected activities and the body weight. Lines represent the [^18^F]UCB-H uptake over a 60 min acquisition after pre-treatment with the vehicle, SV2A_L_, SV2B_L_, or SV2C_L_. (**B**) The bar plots represent the AUC in the eight ROIs (mean ± SEM, n = 5).

In Figure 3A, we can observe the time activity curves (TACs) corresponding to the four different treatments (vehicle, SV2A_L_, SV2B_L_ and SV2C_L_), for one of the regions of interest (ROIs): the whole brain. The four TACs reveal a high initial uptake of [^18^F]UCB-H, which peaks around 5 min post-injection. Subsequently, the radioactivity is quickly washed out of the brain. Some differences can be observed in the kinetic of the TAC after pre-treatment with the respective ligands: The highest peak activity is observed after pre-treatment with the vehicle and with SV2C_L_. Interestingly, the pre-treatments with SV2A_L_ and SV2B_L_ display the same peak of initial uptake. In the case of SV2B_L_, the kinetics of the TAC from 15 to 60 min are similar to the kinetics of the radiotracer after pre-treatment with either the vehicle or SV2C_L_. The TACs for all the ROIs are included in Supplementary Figure S1. In addition, the area under each TAC (the AUC) is represented in Figure 3B, where we can observe the differences between the [^18^F]UCB-H uptake after pre-treatment with SV2A_L_, and after pre-treatment with the other compounds.

**Figure 4:**
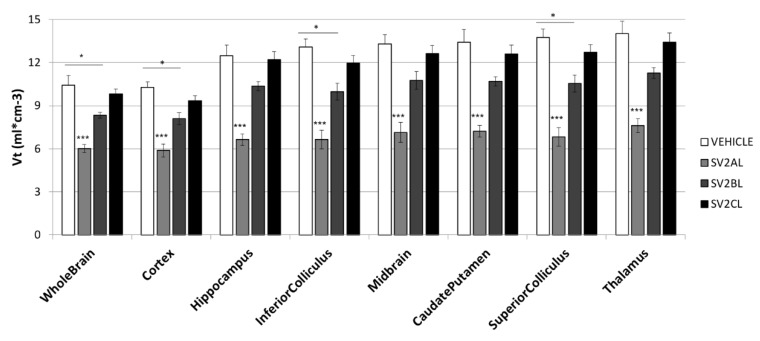
Vt values for the eight selected brain structures. Bars represent the mean ± SEM (n = 5). One-way ANOVA and Scheffe post-hoc tests were performed, with ***p < 0.001 and *p < 0.05.

Figure 4 presents the mean Vt values for the eight selected brain structures, calculated from the previous TACs and the population-based input function (PBIF). Comparing Figure 3B and Figure 4, the differences between groups in AUC and in Vt are similar, with the highest value associated to pre-treatment with the vehicle, and the lowest value for pre-treatment with SV2A_L_.

Table 2 summarizes, for the same regions, the impact on the Vt induced by the blocking experiments, expressed as the relative difference in Vt between the vehicle group and the pre-treated groups. In the whole brain, mean Vt values of 10.4 ± 0.7, 6.0 ± 0.3, 8.3 ± 0.2 and 9.8 ± 0.3 were obtained for the vehicle (control) group, the SV2A_L_ pre-treated group, the SV2B_L_ pre-treated group and the SV2C_L_ pre-treated group, respectively. For the eight ROIs, a statistically significant difference was observed between the SV2A_L_ pre-treated group and all the other groups (*p* < 0.001). Furthermore, for the whole brain, the cerebral cortex and the inferior and superior colliculus, a statistically significant difference was also detected between the vehicle group and the SV2B_L_ pre-treated group (*p* < 0.05).

## 3. Discussion

The SV2 protein is critical for the adequate functioning of the central nervous system, acting as a modulator of synaptic transmission by priming vesicles in quiescent neurons [4]. The divergent roles of the three isoforms which comprise this family have yet to be clarified, although different pathologies have been associated with them. As previously stated, the SV2A isoform is associated with the physiopathology of epilepsy [5,27]. In contrast, the SV2B isoform is related with prostate small cell carcinoma [28] and the SV2C isoform is generally associated with the correct functioning of basal ganglia nuclei [9,29,30]. Some studies have evaluated the possible relation between SV2C and Parkinson’s disease, as SV2C modulates dopamine release [29,31].

This paper’s goal was to evaluate, for the first time, in vivo the specificity of the [^18^F]UCB-H radiotracer in targeting the SV2A isoform compared to SV2B and SV2C. The relevance of such a study stems from the fact that in vivo SV2A quantification can be considered to be an indirect measure of the synaptic density [2,3,18], which is a key parameter for fundamental research and for the clinic.

Before discussing the results obtained during these blocking experiments, we have to address some general considerations. Firstly, the results presented issue from the microPET imaging technique. Like many other microPET cameras, the Focus120 used during this work has a spatial resolution of 1.5 mm, at best hampering the study of brain structures of small size due to the partial volume effect [32,33]. Secondly, the rat brain distributions of the three SV2 protein isoforms [7,8,9] indicate that almost all major brain structures express at least two SV2 protein isoforms. SV2A, the most extensively studied, is ubiquitously distributed [1,8]. Like SV2A, SV2B can be found in almost all the rat brain structures with few subtle differential expressions in some hippocampal substructures like CA3 and the dentate gyrus, along with the reticular nucleus of the thalamus and some small areas in the brain stem [8,9,34]. Unfortunately, these regions are far too small to be correctly quantified with microPET. Janz and Sudhof showed that unlike SV2A and SV2B, the SV2C protein is characterized by much more restricted localization in brain regions considered to be evolutionarily well preserved in rats: The olfactory bulb, the striatum, the substantia nigra, and some nuclei in the pons and the medulla oblongata [9]. As we can see, it is impossible to find well defined brain structures for in vivo microPET quantification in which one of the three isoforms is uniquely or even mostly expressed. Another important point is that we do not have a clear picture of the respective proportions of each isoform present in the main rat brain structures. All these considerations will have to be taken into account in the following discussion. Accordingly, we have decided to select eight major ROIs to ensure robust in vivo quantification with microPET: Whole brain, cortex, hippocampus, inferior colliculus, superior colliculus, midbrain, caudate putamen and thalamus.

The [^18^F]UCB-H Vt values obtained during this study for the vehicle pre-treated group, calculated using the PBIF [35] were in good agreement with those previously published for rats [35,36]. This is important in order to establish the consistency of the proposed methodology. The blocking experiments realized with SV2A_L_ at 10 mg/kg demonstrated a clear significant competition (46.2%) between levetiracetam and [^18^F]UCB-H in eight selected ROIs. These values are of the same order of magnitude as those previously reported in rats [36]. According to the potency of levetiracetam for SV2A against SV2B and SV2C (Table 2), we can conclude that SV2A is one of the main target of [^18^F]UCB-H in vivo in rats.

After performing a blocking experiment with SV2C_L_ at 3 mg/kg, we obtained TACs with similar peaks and kinetics to the TAC corresponding to pre-treatment with the vehicle, in all ROIs. The quantification of the radiotracer uptake, using the Vt, highlighted no statistically significant in vivo competition between SV2C_L_ and [^18^F]UCB-H in any of the eight selected ROIs. From this we can infer that SV2C_L_ pre-treatment has either no impact or an impact of very small size. The population used (n = 5) is not sufficient to demonstrate an effect of small size (f = 0.10), but is optimal to detect medium (f = 0.25) and large effect sizes (f = 0.5). Another important point is that a highly potent SV2C competitor like UCB0949 (pIC_50_ of 7.8) was unable to modify [^18^F]UCB-H binding in brain structures with a high expression of SV2C, like the midbrain or the caudate/putamen [9]. The reduction measured in these regions was of the same order of magnitude as that found in the other structures. These considerations support the theory that SV2C does not seem to be the main target of [^18^F]UCB-H in rats.

The pre-treatment with SV2B_L_ at 3 mg/kg resulted in a TAC with a peak as high as that obtained after pre-treatment with SV2A_L_. However, it features kinetics which are similar to those obtained after pre-treatment with the vehicle or SV2C_L_. This lower peak could be attributed to an initial non-specific binding of SV2B_L_ to the SV2A protein, for which it presents an affinity which is similar to that of SV2A_L_, with a pIC_50_ = 5.6. After the peak, the SV2B_L_ TAC follows a similar shape and level to those of pre-treatment with the vehicle and SV2C_L_, indicating a washing out effect of this fraction of non-specific binding of SV2B_L_ to the SV2A protein. In order to confirm this hypothesis, a similar experiment with a SV2B_L_ with a lower affinity for SV2A should be performed. However, the SV2B_L_ used in this paper is currently the only one available. In addition to the previous analysis, we evaluated the changes in Vt after pre-treatment with SV2B_L_. In these results, we can observe a consistent mean reduction of 20.5% of the Vt values in the eight selected ROIs. The SV2B_L_ is characterized by a pIC_50_ of 7.8 for the SV2B isoform. Such a highly potent competitor is expected to effectively impede the binding of any radioligand to the SV2B isoform. If SV2B was the main target for [^18^F]UCB-H, the blocking induced with this highly efficient SV2B_L_ would have been very pronounced and much higher than the 20% measured. In order to explain the 20% reduction of [^18^F]UCB-H Vt values, we have to take into account that SV2B_L_ also presents some potency for SV2A. SV2B_L_ has a pIC_50_ of 5.6, which is of the same order of magnitude as that of levetiracetam. Thus, the SV2B_L_ ligand has some affinity for SV2A, which could lead to partial blocking of SV2A. Hence, the 20% reduction observed is most likely linked to SV2A blocking induced by SV2B_L_. Accordingly, we can conclude that SV2B does not seem to be the main target of [^18^F]UCB-H in vivo in rats.

We are aware that the respective affinities of SV2B_L_ and SV2C_L_ are a problem for the interpretation of the data, but we have to consider that today UCB5203 and UCB0949 are the only compounds that can be used for this purpose.

## 4. Materials and Methods

### 4.1. Animals

Twenty male Sprague Dawley CD rats (five weeks old) were used, bred by Janvier Laboratories (France). The animals were housed in pairs for three weeks under standard 12:12 h light/dark conditions, maintaining room temperature at 22 °C, and humidity at approximately 50%. Standard pellet food and water were provided ad libitum.

The experimental procedures and protocols used in this investigation (“Synap-SV2A project” files 14-1753 and 13-1573) were reviewed and approved by the Institutional Animal Care and Use Committee of the University of Liege, according to the Helsinki declaration, and conducted in accordance with the European guidelines for care of laboratory animals (2010/63/EU). Moreover, the Animal Research Reporting In Vivo Experiments (ARRIVE) guidelines [37] were followed as closely as possible to confer a minimal intrinsic quality to the study.

### 4.2. Radiopharmaceutical Production and Drugs

[^18^F]UCB-H was produced through one-step radiolabeling of a pyridyliodonium precursor. This method provides 34% ± 2% of injectable [^18^F]UCB-H (uncorrected radiochemical yield) from up to 285 GBq (7.7 Ci) of [^18^F]fluoride (specific activity of 815 ± 185 GBq/μmol and measured purity of 99.8 ± 0.5 wt %); this has previously been reported in Warnier et al. [17].

The ligand for the SV2A isoform (SV2A_L_) was purchased as an injectable solution (levetiracetam, Keppra^®^, UCB Pharma S.A. Brussels, Belgium). At the present time, there are no commercially available specific ligands for the other two SV2 isoforms (SV2B and SV2C). The competitors used were obtained from UCB Pharma s.a.: UCB5203 for SV2B (SV2B_L_, MW: 236.238 g/mol) and UCB0949 for the SV2C (SV2C_L_, MW: 281.197 g/mol). The information on these compounds was supplied by UCB Pharma s.a. The respective affinities for the different SV2 isoforms are presented in Table 2.

The competitors were prepared daily in a vehicle composed of distilled water containing 1% methyl cellulose (viscosity: 15 cP, Sigma-Aldrich, Overijse, Belgium) and 5% dimethyl sulphoxide (DMSO, Sigma-Aldrich, Belgium). The concentrations differed depending on the product specifications and their respective pharmacokinetics, provided by UCB Pharma s.a. The dosing used was 10 mg/kg for SV2A_L_, 3 mg/kg for SV2B_L_, and 3 mg/kg for SV2C_L_. All solutions were administered through the intraperitoneal (i.p.) route in a total volume of 1 mL per kg of body weight. The animals used as a control group (vehicle) received an equal volume of vehicle through the same route of administration.

### 4.3. In Vitro Binding Assays

Reagents and reference compounds used were of analytical grade and obtained from various commercial sources. All cell culture reagents were obtained from Invitrogen (Merelbeke, Belgium). Radioligands (3H-UCB30889, 1184 GBq/mmol; 3H-UCB1418435, 925 GBq/mmol; and 3H-UCB101275-1, 1110–1480 GBq/mmol) were obtained from G.E Healthcare, Amersham, UK (now Perkin Elmer, Zaventem, Belgium) and reference compounds (levetiracetam, UCB108649-1 and UCB101275-1) were custom synthesized and stored according to manufacturer’s recommendations. Test and reference compounds were dissolved in 100% DMSO or H_2_O to give 1 or 10 mM stock solution. The final DMSO concentration in assays was 0.1% unless otherwise stated.

Cell lines generated at UCB Biopharma were human embryonic kidney (HEK) 293 cells expressing human SV2A, SV2B or SV2C proteins. Cells were cultured in Dulbecco’s Modified Eagle medium. The culture medium was supplemented with foetal bovine serum (FBS, 10%), 2 mM l-glutamine, 50 to 100 U/mL penicillin, 50 to 100 µg/mL streptomycin, and 200 µg/mL hygromycin B. Cells were grown at 37 °C with 95% air. Confluent cells were detached by 10 min incubation at 37 °C in phosphate buffered saline (PBS) containing 0.02% EDTA. Culture flasks were washed with 15 mL of ice-cold PBS. The cell suspension was centrifuged at 1500× *g* for 10 min at 4 °C. The pellet was homogenized in 15 mM Tris-HCl buffer (pH 7.5) containing 2 mM MgCl_2_, 0.3 mM EDTA, and 1 mM EGTA (buffer A) using a glass/teflon homogenizer. The crude homogenate was subjected to a freeze and thaw cycle in liquid nitrogen and DNAse (1 µL/mL) was then added. The homogenate was further incubated for 10 min at 25 °C before being centrifuged at 40,000× *g* for 25 min at 4 °C. The pellet was re-suspended in buffer A and washed once under the same conditions. The final crude membrane pellet was re-suspended at a protein concentration of 1–3 mg/mL in 7.5 mM Tris-HCl buffer (pH 7.5 at 25 °C) containing 250 mM sucrose and stored in liquid nitrogen until use.

Membranes were incubated in binding buffer (see Table 3) containing test compound or positive control in the presence of the radioligand. The non-specific binding (NSB) was defined as the residual binding observed in the presence of a high concentration (1000 fold its Ki) of a specific unlabeled reference compound. Membrane-bound and free radioligands were separated by rapid filtration through glass fiber filters (GF/C). Samples and filters were rinsed using at least 6 mL of washing buffer. The entire filtration procedure did not exceed 10 s per sample. The radioactivity trapped on the filters was counted by liquid scintillation in a β-counter. To determine the affinity of a compound for a given target, competition curves were performed with at least 10 concentrations of compound spanning at least 5 log units.

### 4.4. PET Acquisitions

The animals (n = 5 per group) were anesthetized using 4% isoflurane in air at a flow rate of 1 L/min during induction and 1.5% to 2% isoflurane in air at 0.6 L/min during maintenance. Respiration rate and rectal temperature were continuously measured using a physiological monitoring system (Minerve, France). The temperature was maintained at 37 ± 0.5 °C using an air warming system.

MicroPET scans were performed with a Siemens FOCUS 120 microPET (Siemens, Knoxville, TN, USA). The animals were anesthetized and pre-treated i.p. with vehicle, SV2A_L_, SV2B_L_, or SV2C_L_. Thirty minutes later, they were installed in the microPET scanner and [^18^F]UCB-H was injected via the lateral tail vein (44.7 ± 3 MBq, 0.55 mL), simultaneously starting a 60 min emission scan, in list mode. Finally, a 10 min transmission scan was performed in a single event acquisition mode, using a ^57^Co source. The acquired data were then reframed as follows: 6 × 5 s, 6 × 10 s, 3 × 20 s, 5 × 30 s, 5 × 60 s, 8 × 150 s, and 6 × 300 s. For each frame, a total of 95 trans-axial slices were obtained using Fourier rebining (FORE), followed by 2D ramp filtered backprojection (FBP), in 256 × 256 matrix. The slice thickness was 0.796 mm and the in-slice pixel size was 0.433 mm.

Immediately after the PET acquisition, the anesthetized rats were transferred into a 9.4 Tesla MRI horizontal bore system (Agilent Technologies, Palo Alto, CA, USA), with a 72 mm inner diameter volumetric coil (Rapid Biomedical GmbH, Würzurg, Germany). Anatomical T2-weighted brain images were obtained using a fast spin echo multi-slice sequence with the following parameters: TR = 2000 ms, TE = 40 ms, matrix = 256 × 256, FOV = 45 × 45 mm, 30 contiguous slices of thickness = 0.80 mm and in-plane voxel size = 0.176 × 0.176 mm.

### 4.5. Imaging Data Processing

PMOD software (Version 3.6, PMOD Technologies, Zurich, Switzerland) was used to process the imaging data. The structural MRI images were firstly co-registered to the corresponding PET images, and subsequently spatially normalized into the PMOD MRI T2 template. Finally, the inverse normalization parameters were calculated and applied to the PMOD rat brain atlas to bring it in the individual PET space. From this atlas, eight relevant regions of interest (ROIs) were chosen according to their differential expression of SV2A, SV2B and SV2C: whole brain, cortex, caudate/putamen, hippocampus, inferior colliculus, superior colliculus, midbrain and thalamus.

Individual time-activity curves (TACs) were extracted for each ROIs and normalized by the body weight and the injected dose of radiotracer to be expressed as standardized uptake value (SUV). A population-based input function (PBIF) published by our laboratory [35] was used to avoid arterial blood sampling during the acquisitions. The distribution volume (Vt), was determined by Logan plot kinetic modelling using the TACs and the PBIF. The equilibration time (t*) was fixed at 15 min (starting point of the range used in the multi-linear regression analysis).

### 4.6. Statistical Analysis

The results are presented as mean (Vt) ± standard error of the mean (SEM). All the data were tested for normal distribution with Levene’s test for homogeneity, and with a Kolmogorov–Smirnov test for normality. Data were analyzed using one-way analysis of variance (ANOVA) followed by Scheffe post-hoc tests.

All statistical analyses were performed with the statistics software Statistica 12 (Statsoft, France) and GraphPad Prism (version 6, GraphPad software, Inc., San Diego, CA, USA). The critical level of statistical significance was always set at *p* < 0.05.

## 5. Conclusions

For the first time, the specificity of a radiopharmaceutical compound for the three SV2 protein isoforms was assessed in vivo, in rats. The results obtained clearly indicated that SV2A was the main target of [^18^F]UCB-H, and confirmed that [^18^F]UCB-H is a suitable radiotracer for in vivo imaging of the SV2A proteins with PET. Consequently, [^18^F]UCB-H is an interesting candidate to study SV2A-associated pathologies.

## Figures and Tables

**Table 1:** pIC_50_ of the competitors used for the different SV2 isoforms. Binding affinities measured for human SV2 proteins at 37 °C. Data are presented as mean (n = 3 to 10) from non-linear regression analysis of raw data using a sigmoidal dose-response model. Additional data for SV2B_L_ (UCB5203) solubility: 0.1 mg/mL, route of administration: ip (suspension in 5% DMSO–1% methyl cellulose in water), CEREP @ 10 µM: all targets < 50% inhibition, mouse brain fraction unbound: 37%, mouse brain exposure (3 mg/kg, 30–60 min): ~1.8 µM total → 0.66 µM free → ~100 fold IC50 SV2B. Additional data for SV2C_L_ (UCB0949) solubility: 0.055 mg/mL, route of administration: ip (suspension in 5% DMSO—1% methyl cellulose in water), CEREP @ 10 µM: all targets < 50% inhibition, mouse brain fraction unbound: 54%, mouse brain exposure (3 mg/kg, 30–60 min): ~8 µM total → 4.3 µM free → ~270 fold IC_50_ SV2C.

	Synaptic Vesicle Protein Isoforms		
	SV2A	SV2B	SV2C
**SV2A_L_**	5.2	−3.1	−3.2
**SV2B_L_**	5.6	7.8	5.5
**SV2C_L_**	<5	5.9	7.8

**Table 2:** Illustration of the impact induced by the blocking experiments, expressed as percentage of reduction calculated from the mean Vt values (n = 5) for the eight selected ROIs.

ROIs	Vehicle vs. SV2A_L_	Vehicle vs. SV2B_L_	Vehicle vs. SV2C_L_
Whole brain	42.3	19.9	5.6
Cortex	42.7	21.1	9.1
Hippocampus	46.8	16.9	2.1
Inferior colliculus	49.1	23.7	8.5
Midbrain	46.3	19.1	5.1
Caudate/Putamen	46.2	20.2	6.2
Superior colliculus	50.4	23.4	7.6
Thalamus	45.9	19.7	4.4
Mean	46.2	20.5	6.1
SEM	1.0	0.8	0.8

**Table 3:** Details of the in vitro binding assay determination. Percentage of inhibition was calculated as follows: % INHIBITION = 100 − [((BI − NSB)/(B0 − NSB)) × 100], where B0 and BI represent the binding observed in the absence and presence of the test compound, respectively (dpm), NSB is the radioligand non-specific binding (dpm). Raw data were analyzed by non-linear regression using XLfit^TM^ (IDBS, London, Great Britain) according to the following generic equation: B = NSB + [(B0 − NSB)/(1 + (((10^X^)/(10^−pIC^^50^))^nH^))], where B is the radioligand bound in the presence of the unlabeled compound (dpm), NSB is the radioligand non-specific binding (dpm), B0 is the radioligand bound in the absence of unlabeled compound (dpm), X is the concentration of unlabeled compound (log M), pIC_50_ is the concentration of unlabeled compound that inhibits the radioligand specific binding by 50% (−log M), and nH is the Hill coefficient.

In Vitro Binding Details	hSV2A Assay	hSV2B	hSV2C
**Binding buffer**	50 mM Tris-HCl (pH 7.4) containing 2 mM MgCl_2_		
**Filtration buffer**	Ice-cold 50 mM Tris-HCl (pH 7.4)		
**Incubation time**	120 min at 37 °C in 0.5 mL	120 min at 37 °C in 0.5 mL	120 min at 37 °C in 0.2 mL
**Radioligand**	^3^H-UCB30889 (4 nM)	^3^H-UCB1418435 (8 nM)	^3^H-UCB101275-1 (20 nM)
**Proteins**	75–125 µg HEK293 membranes	2–5 µg HEK293 membranes	40–60 µg HEK293 membranes
**Blocking drug**	Levetiracetam (1 mM)	UCB108649-2 (10 µM)	UCB101275-1 (100 µM)

## Supplementary Materials

The following are available online. Figure S1: TACs extracted from the eight ROIs, and normalized by the injected activities and the body weight. Lines represent the [^18^F]UCB-H uptake over a 60 min acquisition after pre-treatment with the vehicle, SV2A_L_, SV2B_L_, or SV2C_L_ (mean ± SEM; n = 5).
