# Supplementary material for: Evaluating the In Vivo Specificity of [18F]UCB-H for the SV2A Protein, Compared with SV2B and SV2C in Rats Using microPET
Source: Molecules. 2019 May 1;24(9):1705. doi: 10.3390/molecules24091705 (PMC6538996; doi:10.3390/molecules24091705)
Supplement: Supplementary file 1 [file molecules-24-01705-s001.pdf]

Supplementary Data

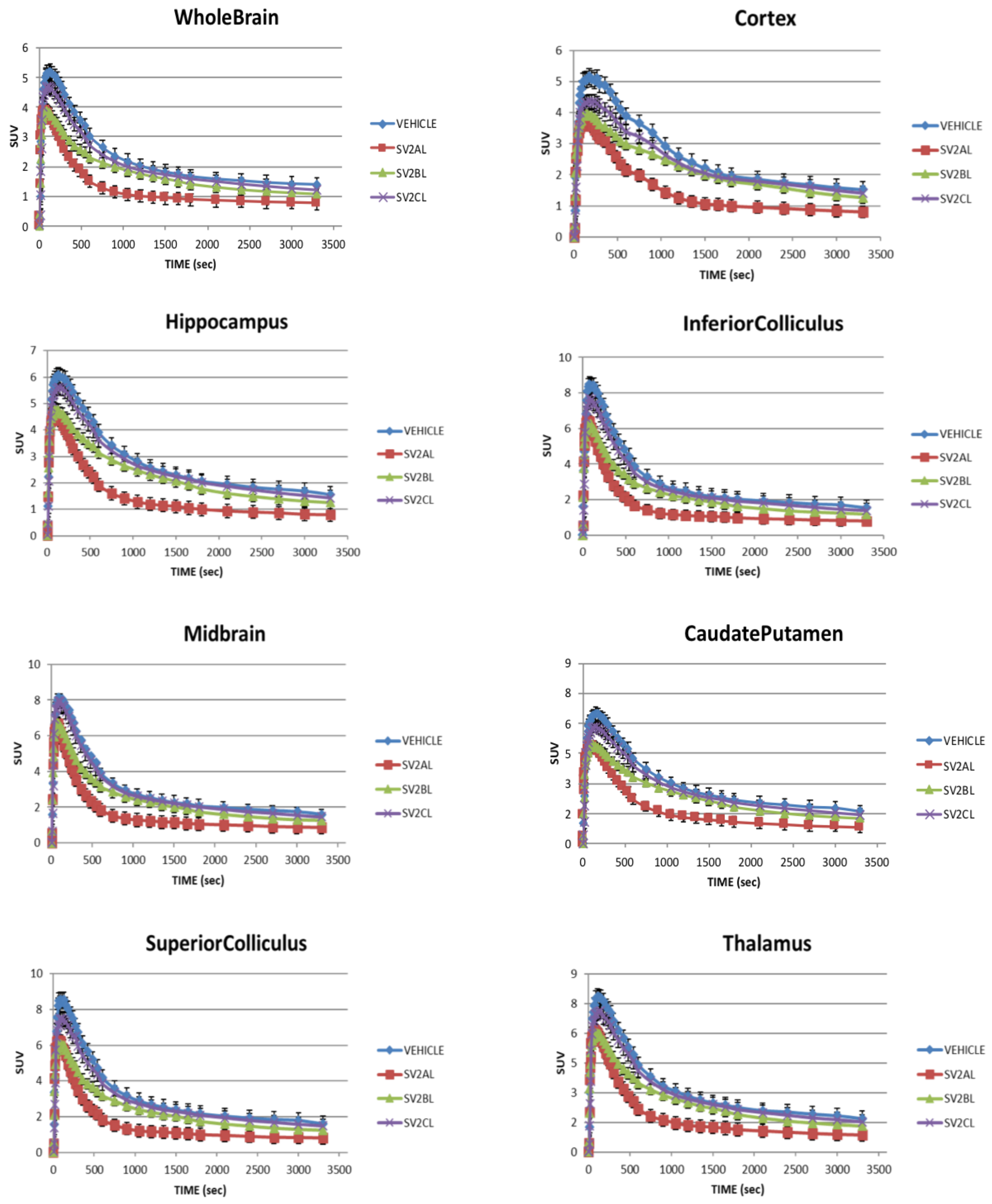

Figure S1. Time activity curves (TACs) corresponding to the four different treatments (vehicle, SV2AL, SV2BL, and SV2CL), for all the regions of interest (ROIs).
